# Drainage Recycling Ratio Influences Yield, Fruit Quality, and Antioxidant Properties of Korean Strawberry ‘Seolhyang’

**DOI:** 10.3390/plants14192984

**Published:** 2025-09-26

**Authors:** Minkyung Kim, M. G. Rabbani, Youngae Jeong, Mewuleddeg Zebro, Jeonghyeon Baek, Ki-Young Choi

**Affiliations:** 1 Department of Agriculture and Industries, Kangwon National University, Chuncheon 24341, Republic of Korea; minkyeong348@kangwon.ac.kr (M.K.); rabbani1572@gmail.com (M.G.R.); ya15168@kangwon.ac.kr (Y.J.); zmewuledeg@gwnu.ac.kr (M.Z.); unoblue@kangwon.ac.kr (J.B.); 2 Department of Horticulture and Plant Science, Jimma University, Jimma 378, Ethiopia; 3 Department of Smartfarm and Agricultural Industry, Kangwon National University, Chuncheon 24341, Republic of Korea

**Keywords:** drainage recycling ratio, semi-closed hydroponics, ionic balance, antioxidant, fruit weight

## Abstract

Closed hydroponic systems for strawberries (*Fragaria* × *ananassa* Duch.) are infrequently used because the crop is highly sensitive to salt accumulation and prone to root diseases, resulting in yield reduction. This study investigated semi-closed hydroponic systems using various drainage recycling ratios (30%, 50%, and 70% of drainage EC) to determine their impact on yield, fruit quality, and antioxidant properties. Recycling at moderate levels (30–50%) effectively maintained ionic balance, particularly with respect to K/N and K/Ca ratios, which enabled stable yields and increased fruit weight similar to the control (open hydroponic system) group. Conversely, a high recycling ratio (70%) led to ionic imbalances—characterized by increased K/N ratios and higher concentrations of Na^+^, Cl^−^, and SO_4_^2−^—that were associated with decreased fruit size. Measures of antioxidant capacity, such as total phenol and flavonoid content, ferric reducing antioxidant power, and DPPH activity, were not significantly influenced by the recycling ratio alone. Nevertheless, the relatively elevated antioxidant activity observed at the 70% recycling level indicates a mild ionic and osmotic stress response likely caused by increased salt concentration. Changes related to the cropping system season, rather than ion variations from recycling, exerted a stronger influence on antioxidant accumulation. In summary, moderate drainage recycling facilitates optimal fruit production without negatively affecting quality, while excessive recycling may increase antioxidant activity but leads to reduced yields. The results provide practical recommendations for optimizing nutrient reuse in semi-closed strawberry hydroponic systems.

## 1. Introduction

Strawberry (*Fragaria* × *ananassa* Duch.) is a widely grown fruit crop worldwide, prized for its distinctive flavor and aroma, as well as its high levels of vitamins, phenolic compounds, and antioxidant activity [1]. In 2023, strawberry global production reached 10.5 million ton, reflecting an average annual growth rate of 3.5% [2]. China led production with 2.1 million ton, followed by the United States of America (1.1 million), and the Republic of Korea ranked tenth with 177 thousand tons [2]. However, the Republic of Korea achieved a higher production level (30.4 t·ha^−1^) compared to China (27.0 t·ha^−1^) and the global average (24.1 t·ha^−1^) [3].

To facilitate year-round cultivation and enhance resource efficiency, hydroponic strawberry production is gaining popularity, particularly within protected environment agriculture. In the Republic of Korea, 28.3% of strawberry crops were cultivated hydroponically in 2020, with this proportion showing a continued upward trend [3]. Hydroponic strawberry cultivation in Korea is typically practiced from September to May, although some regions also conduct summer cropping from April to October. However, strawberries exhibit physiological sensitivity to ionic imbalances and salinity stress, which is attributed to their shallow root architecture and considerable requirements for precise management of water and nutrients [4,5]. As a result, they are less compatible with closed hydroponic systems, where ongoing recirculation of nutrient solutions can promote salt build-up and subsequently decrease productivity [6].

In recent years, semi-closed hydroponic systems have been examined in the Republic of Korea as a potential middle ground between sustainability and adequate plant safety; in these systems, part of the drainage is recycled based on electrical conductivity (EC) setpoints [7,8]. While such systems demonstrate potential to reduce both water and fertilizer usage and decrease environmental discharge. In the system, the percentage of drainage to be recycled, known as the drainage recycling ratio, is critical in achieving the benefits of reuse without imposing negative outcomes. An overly aggressive recycling strategy may improve resource use efficiency but increases the likelihood of inducing physiological stress and yield loss, whereas excessively cautious reuse could limit the progress toward sustainable practice.

Selecting an optimal recycling ratio supports appropriate ion concentrations in the root zone conducive to plant growth, while an excessive ratio can result in the gradual accumulation of salts—particularly those ions, including Na^+^, Cl^−^, and SO_4_^2−^, that are poorly absorbed by plants [9]. These ions may interfere with nutrient absorption through competitive inhibition or by modifying the osmotic environment, affecting processes such as transpiration, calcium mobility, and root-zone pH [10]. For instance, increased Cl^−^ concentrations have been shown to diminish nitrate uptake, and disproportionately high K^+^ compared to Ca^2+^ can compromise fruit firmness and heighten the strawberry crop’s vulnerability to physiological disorders [11,12]. Therefore, careful management of the drainage recycling ratio is required to achieve a balance between maximizing resource efficiency and maintaining both productivity and fruit quality.

Research in paprika has established that recycling 20–50% of drainage based on EC can significantly reduce nutrient discharge while maintaining yield [7,13]. In contrast, strawberries exhibit greater sensitivity to salt stress and have lower ion exclusion capacity, potentially requiring stricter control of recycling thresholds. Regular adjustment of the nutrient solution according to the nutrient contents detected in drainage, or complete renewal of the nutrient solution, is recommended to prevent ionic imbalance and avoid reductions in fruit size or yield in berry crops [9]. Simultaneously, mild abiotic stress—such as moderate salinity—has been observed to enhance the accumulation of antioxidant compounds, including phenolics and flavonoids, in strawberries [14,15]. Although this may improve the nutritional quality of the fruit, it may also indicate stress-related metabolic changes that could negatively impact yield performance. These physiological trade-offs emphasize the complexity involved in optimizing drainage reuse within hydroponic strawberry production. Therefore, gaining insight into the physiological responses of strawberries to different recycling ratios in the absence of nutrient adjustments is essential for designing semi-closed systems that are productive and environmentally sustainable.

Although sustainable hydroponics is attracting increasing interest, studies remain limited on how varying drainage recycling ratios influence the interrelationship among yield, fruit quality, antioxidant attributes, and ionic dynamics in strawberries. Accordingly, this study was designed to investigate the effects of different drainage recycling ratios on yield, fruit quality, antioxidant activity, and ionic balance in semi-closed hydroponic strawberry cultivation without nutrient solution adjustment. Special focus was given to the influence of critical nutrient ratios (e.g., K/N and K/Ca) and the potential trade-offs that may arise between productivity and physiological stress responses.

## 2. Results and Discussion

### 2.1. Inorganic Ionic Contents in Supplied Nutrient Solution

Inorganic ionic concentrations in the supplied nutrient solution exhibited significant variation depending on both the month of cultivation and the drainage recycling ratio treatment (Table 1). For nitrogen forms, ammonium (NH_4_^+^) concentrations consistently decreased throughout the growing season under all treatments (*p* ≤ 0.001), indicative of rapid root uptake and possible nitrification within the rhizosphere. Nitrate (NO_3_^−^) reached its lowest value in January, following the vegetative phase, before gradually rising in February and March, corresponding with the period of concurrent vegetative and reproductive growth. Phosphate (PO_4_^3−^) and potassium (K^+^) concentrations increased significantly over time (*p* ≤ 0.001), particularly due to the application of an additional 20% potassium phosphate fertilizer initiated in January.

Nonetheless, concentrations of NO_3_^−^, NH_4_^+^, PO_4_^3−^, and K^+^ generally declined as drainage recycling ratios increased from January to March, indicating depletion from repeated recirculation and plant uptake. In contrast, sodium (Na^+^) and chloride (Cl^−^) accumulated progressively within the recycled solutions. Notably, Na^+^ concentrations in D70% (nutrient solution recycled at 70% of the drainage EC) were elevated by 100–188% compared to the control throughout January to March, which highlights poor selectivity for Na^+^ during recycling; this is recognized to disrupt nutrient balance and osmotic potential in conditions of high accumulation [10]. Although calcium (Ca^2+^), magnesium (Mg^2+^), and sulfate (SO_4_^2−^) concentrations remained relatively stable overall, their levels tended to rise with higher recycling ratios, especially for SO_4_^2−^ in D70% by March. These ions, particularly Ca^2+^ and Mg^2+^, are less mobile in solution and accumulate more gradually, which may account for their incremental increase during extended recycling periods [16].

Total ionic concentrations—summing all cations and anions—remained relatively constant across treatments, with averages around 9.7–11.2 mEq·L^−1^. The ionic balance, measured by the cation-to-anion (C/A) ratio, displayed clear effects of the treatment conditions. Under higher recycling ratios, the C/A ratio exhibited pronounced deviations. From January to March, D50% and D70% treatments showed elevated cation ratios compared to the control and D30%, with values exceeding 1.5 and a maximum of 1.75 recorded in D50% in January. These increased ratios indicate either a reduction in the availability of key anions (e.g., NO_3_^−^, PO_4_^3−^) or the accumulation of non-essential or slowly absorbed cations (e.g., Na^+^, Ca^2+^).

Significant interactions between month and recycling treatment suggest that prolonged recycling at high ratios (D70%) exacerbated ionic imbalances over time. This finding indicates that while moderate recycling ratios (D30–50%) may facilitate sustainable nutrient reuse, excessive recycling (D70%) leads to disproportionate accumulation of specific ions—particularly Na^+^, Cl^−^, and SO_4_^2−^—and disrupts ionic equilibrium, potentially hindering nutrient absorption or triggering stress responses in plants [17].

**Figure 1:**
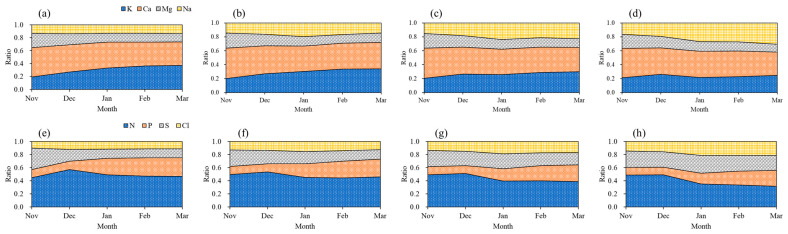
Cation and anion ratios for Control (**a**,**e**), D30% (**b**,**f**), D50% (**c**,**g**), and D70% (**d**,**h**) nutrient solutions in a closed hydroponic system for strawberry cultivation under various drainage recycling ratios.

Monthly variation in the cation and anion composition of the nutrient solution for different drainage recycling ratio treatments exhibited pronounced changes in both ratio and trend by month and treatment (Figure 1). In the Control and D30% treatments, the ionic profile remained relatively consistent among the months, with NO_3_^−^ and K^+^ persistently composing the majority. However, in the D50% and especially D70% treatments, there were progressive increases in Na^+^ and Cl^−^ levels, predominantly from January to March, indicating the accumulation of salts due to increased drainage recycling. The percentages of NO_3_^−^ and K^+^ decreased over time in both D50% and D70% treatments, implying a progressive reduction in these essential nutrients as Na^+^ and Cl^−^ became more prominent. Ca^2+^ levels remained moderately unchanged across all treatments, but their proportion relative to total ions was reduced in the higher drainage recycling conditions. Collectively, these findings demonstrate that increased recycling ratios (D50–D70%) result in salt accumulation and disrupt the ionic equilibrium in the nutrient solution, with potential adverse effects on nutrient absorption and strawberry fruit quality.

**Figure 2:**
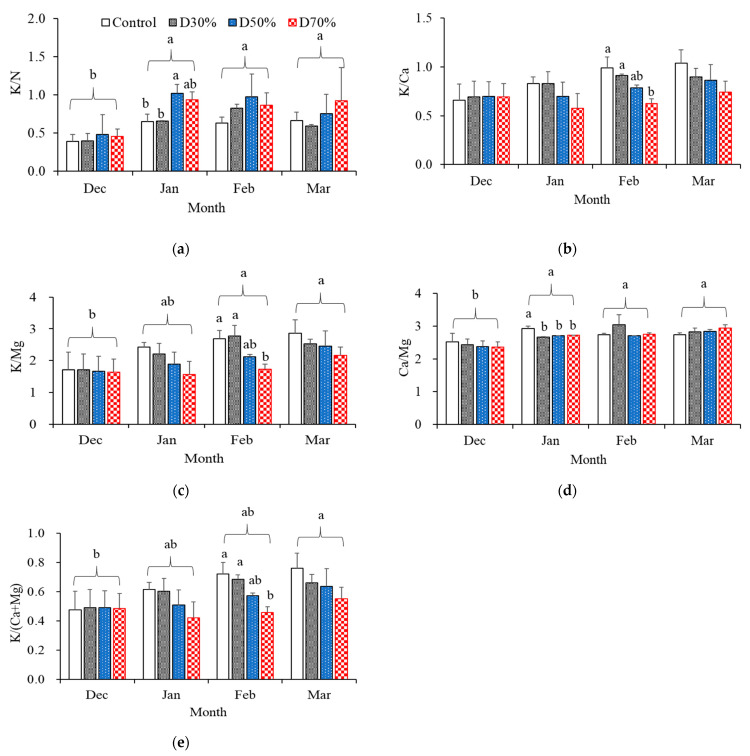
Monthly changes in the K/N (**a**), K/Ca (**b**), K/Mg (**c**), Ca/Mg (**d**), and K/(Ca + Mg) (**e**) of the nutrient solution under four drainage recycling treatments: Control (open), D30% (30% of drainage), D50% (50%), and D70% (70%). Bars represent means ± SD (n = 3). Different letters within each month indicate significant differences among treatments, and among months based on Duncan’s multiple range test (*p* ≤ 0.05).

Temporal shifts in the ionic ratios of nutrient solutions differed significantly based on drainage recycling treatments (Figure 2a–e). The K/N ratio was notably affected by both treatment and sampling month (Figure 2a). During December, K/N ratios were similar among all treatments, recorded at 0.4 to 0.6. Yet, in January, this ratio rose sharply for the D50% and D70% groups, reaching values of 1.1 and 1.0, attributed to a pronounced decrease in nitrate levels compared to potassium. As the crop transitioned from the vegetative stage, which requires high nitrogen, to the harvesting stage, which requires high potassium, in January, the K/N ratio was strongly affected. By March, the K/N ratio persisted at 1.0 for the D70% treatment, while stabilizing at lower levels (0.7–0.8) in the control and D30% treatments, reflecting sustained nitrogen equilibrium in those groups.

For the K/Ca (Figure 2b), all treatments exhibited similar values in December (approximately 0.7), reflecting a balanced supply of potassium and calcium. Over the experimental period, the ratio increased in the control, D30%, and D50% treatments, reaching up to 1.0 and 0.9 by March, respectively, while in the D70% treatment, it remained approximately constant at 0.7. This pattern indicates relatively greater depletion of K^+^ compared to Ca^2+^ when high recycling levels are applied. The K/Mg ratio (Figure 2c) also showed changes based on treatment. In December, all treatments presented similar ratios (1.6–1.7), but starting in January, the ratio increased more markedly in the control and D30% treatments than in D50% and D70%. By March, K/Mg had risen to 2.9 in the control, 2.5 in both D30% and D50%, and 2.2 in D70%, suggesting that Mg^2+^ was depleted or accumulated at a slower rate than K^+^ under higher recycling conditions. These changes are likely attributable to differences in ion uptake efficiency and cation competition, with the effects becoming particularly evident in February for both K/Ca and K/Mg ratios. Conversely, the Ca/Mg remained largely unchanged across treatments and over time (Figure 2d). All groups kept values within the optimal 2.5–2.9 range from December to March, with values of 2.8 (D30% and D50%) and 2.9 (D70%) reported in March. This overall stability indicates comparable uptake or retention behaviors for Ca^2+^ and Mg^2+^, regardless of the recycling ratio applied. The consistent Ca/Mg contrasts with the notable changes seen in K/Ca and K/Mg, potentially representing the lower competitive effects between these two divalent cations under the specific nutrient conditions studied.

To evaluate the predominant status of K^+^ over divalent cations, the K/(Ca + Mg) ratio was determined (Figure 2e). In December, all treatments had similar K/(Ca + Mg) values (~0.4), indicating balanced proportions among potassium, calcium, and magnesium. However, beginning in January, the ratio increased in all groups except for D70%, where it consistently remained below 0.6. By March, the ratio reached its highest value of 0.7 in the control, 0.6 in both D30% and D50%, with D70% staying lowest at 0.5. These trends demonstrate that higher recycling conditions promote accumulation or reduced uptake of Ca^2+^ and Mg^2+^ relative to K^+^, resulting in consistently suppressed K/(Ca + Mg) ratios, which may disrupt nutrient balance. Since K^+^, Ca^2+^, and Mg^2+^ share root transport systems and compete for uptake, it is essential to maintain optimal K/(Ca + Mg) ratios to prevent nutrient antagonism and achieve efficient cation acquisition [10]. Collectively, this analysis of ionic ratio variation indicates that increasing levels of drainage significantly modify nutrient solution composition, particularly the relationship between potassium and both nitrogen and divalent cations. Resultant imbalances such as lowered K/(Ca + Mg) with increased recycling may reduce nutrient bioavailability and thereby negatively impact plant growth and fruit development in extended semi-closed hydroponic cultivation.

Collectively, these findings suggest that increasing the drainage recycling ratio leads to alterations in critical ionic balances within the nutrient solution, with particular impact on potassium and nitrate levels. Changes in K- and N-related ion ratios could influence the efficiency of nutrient uptake and may ultimately impact fruit quality in long-term semi-closed hydroponic systems.

### 2.2. Strawberry Productivity

**Figure 3:**
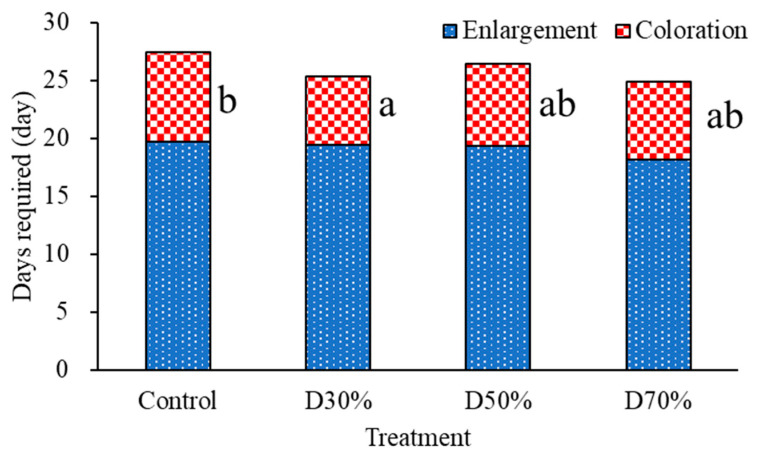
Days required for enlargement and coloring of strawberry fruits from the third truss (From 24 February to 31 March) of strawberries grown in a closed hydroponic system with different drainage recycling ratios. Boxes with different letters are significantly different, and without any letters are not significantly different according to Duncan’s multiple range test at *p* ≤ 0.05 (n = 13).

The duration from fruit set to enlargement and the final coloration period (defined as the interval between the end of fruit enlargement and harvest) of the third truss (late February) of strawberries displayed trends related to the applied treatments; however, not all observed differences reached statistical significance (Figure 3). The fruit enlargement phase was about 19 days across all treatments, indicating that the fruit maturation rate remained relatively unaffected by the drainage recycling ratio. In contrast, more pronounced variation was found in the coloration period among treatments. The control treatment resulted in the longest coloration period (7.8 days), whereas strawberries in the D30% treatment had the shortest coloration period (5.9 days), which was significantly shorter than that of the control (*p* ≤ 0.05). D50% and D70% treatments exhibited intermediate durations, with no significant difference identified compared with either D30% or the control group. The reduced coloration period in D30% may be attributable to modest changes in ripening dynamics, possibly related to moderate ionic alterations. Overall, the total duration from fruit set to harvest remained largely stable despite changes in drainage recycling, yet greater variability in the final coloration phase among treatments was observed. These findings underscore the necessity of closely monitoring key ripening stages to optimize nutrient recycling protocols for quality assurance and precise harvest planning in semi-closed hydroponic strawberry cultivation.

**Figure 4:**
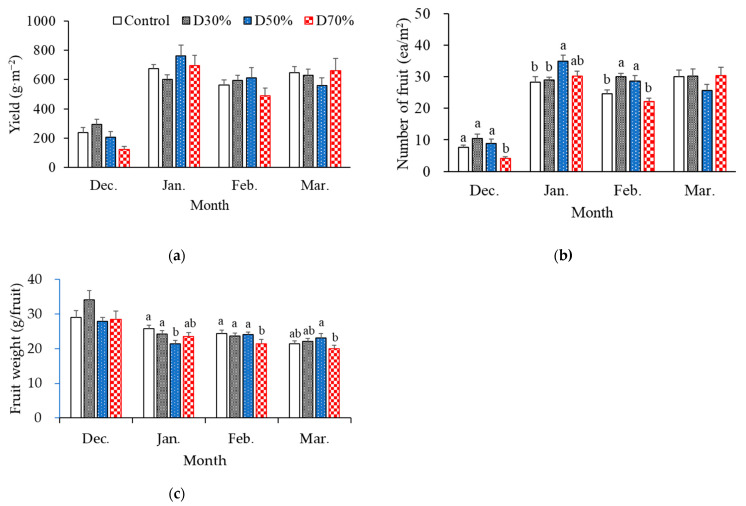
Yield (**a**), number of fruits harvested (**b**), and fruit weight (**c**) of strawberries grown in a semi-closed hydroponic system with different drainage reuse ratios for nutrient solutions. Bars with different letters are significantly different, and without any letters are not significantly different according to Duncan’s multiple range test at *p* ≤ 0.05 (n = 10, where n represents a slab).

Monthly fruit yield and the number of harvested fruits per square meter showed similar seasonal patterns across all drainage recycling treatments (Figure 4a,b), whereas average fruit weight steadily decreased over time (Figure 4c). Both yield and fruit number reached their lowest values in December, which was mainly attributable to shortened harvest periods at the beginning of the season (starting 23 December). During the principal harvesting period from January to March, yield and fruit count increased considerably and remained largely stable.

When comparing treatments, the D50% treatment consistently achieved higher yields than the other treatments during January and February. Conversely, in March, D70% treatments resulted in higher yields compared to the other treatments. The fruit number followed a similar pattern, though differences among treatments were less marked. Across the months, drainage recycling ratios did not significantly impact overall yield or fruit number, even though the D30% and D50% treatments tended to sustain productivity at levels similar to or exceeding the control. The D70% treatment, while not always producing fewer fruits, yielded relatively lower harvests during several months.

Fruit weight decreased from 28–34 g in December to 20–23 g in March for all treatments. This seasonal reduction aligns with the typical decline in berry size as the plant progresses from early to late harvest [18,19,20]. Among the treatments, the D70% consistently produced the smallest fruits throughout the cropping period, most notably in February (21.4 g) and March (20.0 g). The reduced fruit weight observed in the D70% treatment suggests that excessive drainage recycling may limit the resources available for fruit expansion.

Taken together, these findings indicate that moderate drainage recycling ratios (D30% and D50%) and control treatments did not adversely affect fruit development and may contribute to improved productivity, potentially by preserving more stable ionic balances. Likewise, moderate salinity (50 mM NaCl) did not negatively influence tomato yield [21]. However, fruit weight was marginally reduced under higher recycling (70%), potentially as a result of nutritional imbalance. Nitrogen (N) plays a critical role in both vegetative and reproductive phases of strawberry development, including root growth and flower bud initiation [22], and inadequate N supply is associated with reduced fruit size [23]. In the high recycling treatment (D70%), frequent nitrate uptake with insufficient replenishment led to a rise in the K/N ratio (Table 1, and Figure 2a), which signifies nitrate depletion—likely curtailing nitrogen availability and ultimately restricting fruit growth and cell enlargement. The temporal patterns of NO_3_^−^ observed in our study—with lowest levels in January and increasing into February and March—reflect the fluctuating nitrogen requirements of fruiting crops such as strawberry [22]. Furthermore, Jiang et al. [24] demonstrated that a lack of K^+^ in nutrient solutions can diminish strawberry fruit coloration and weight, even when total yield is not markedly affected—highlighting the importance of maintaining appropriate ionic balance and nutrient composition.

Fruit quality attributes were impacted more by seasonal progression than by the drainage recycling treatments, as harvest month significantly affected most quality parameters (Table 2). Fruit length displayed a temporal pattern similar to that of fruit weight, with values declining over the observed period. In December, D70% resulted in fruits approximately 8–10% shorter (45.6 mm) compared to those in the Control (49.5 mm) and D30% (51.3 mm) treatments. By March, the treatment differences narrowed somewhat, but D70% continued to yield the shortest fruits (43.5 mm), compared with 45–46 mm in other treatments. In contrast, fruit width remained largely unchanged, with no distinct tendencies across months or treatments. Neither total soluble solids (TSS) nor titratable acidity (TA) differed significantly by treatment, though month of harvest led to limited fluctuations. TSS was consistent from December through February (12.6–13.4 °Brix), but dropped to about 10.5–11.3 °Brix in March, likely due to rising temperatures [25]. Despite the decrease in TSS, the TSS/TA ratio did not show significant variation, remaining within a range of 17.9 to 27.4, thus indicating relative consistency in perceived sweetness.

Overall, fruit quality characteristics were predominantly affected by harvest month rather than by the direct impact of drainage recycling, although the recycling treatments modified the ionic environment. The D70% treatment, however, presented minor disadvantages, especially regarding fruit length, whereas D30% and D50% consistently promoted balanced fruit quality attributes, such as optimal fruit size and TSS/TA. These results indicate that moderate recycling effectively supports both yield and quality, while an excessively high recycling rate can progressively impair fruit development.

### 2.3. Antioxidant Contents in Strawberry Fruits

Key antioxidant indicators—including total phenol content (TPC), total flavonoid content (TFC), ferric reducing antioxidant power (FRAP), and DPPH activity—were chiefly affected by the month of harvest rather than by recycling ratio (Table 3). All antioxidant markers demonstrated substantial variation over time (*p* ≤ 0.001). TPC (from 24–28 to 27–32 mg) and FRAP (from 0.98–1.28 to 1.20–1.47 mM) progressively increased from December through March, while DPPH activity remained elevated from December to February (75–88%) but declined markedly in March (48–57%), indicating a possible change in antioxidant composition or scavenging activity in late-season fruit. TFC exhibited more inconsistent seasonal trends. While isolated differences among treatments appeared in certain months, no consistent or statistically significant effects due to the drainage recycling ratio were apparent across the antioxidant parameters.

These results—the progressive elevation in TPC and FRAP from December to March, followed by a pronounced reduction in DPPH activity observed in March—indicate seasonal modulation of the antioxidant profile and scavenging capacity in late-season fruit. These trends likely arise from physiological changes associated with ripening, coupled with environmental factors such as variations in light exposure and temperature. Our data are consistent with previous findings that environmental conditions have a more pronounced effect on antioxidant-related properties in strawberries than fertilizer practices. Agehara and Nunes [26] found that supplemental nitrogen fertilization during early vegetative growth increased marketable yield by 18%, but that sugar and antioxidant levels were more substantially impacted by greater light exposure and lower temperatures. Likewise, Cardeñosa et al. [27] detected no significant influence of nitrogen fertilizer type on sugar content, titratable acidity, skin color, or antioxidant content; however, they reported that fruit harvested in January contained more sugar, while those collected in March had higher total phenol and anthocyanin concentrations. Aguero et al. [28] similarly demonstrated that fertilizer inputs exert minimal effects on antioxidant quality, whereas temperature and rainfall strongly influence attributes such as firmness, skin color, titratable acidity, and sugar content. The observed March peak in TPC and FRAP in our study aligns with prior evidence that intensified solar radiation and elevated temperatures promote phenolic metabolism by activating the phenylpropanoid pathway, which increases anthocyanin synthesis [29]. Collectively, these observations support the conclusion that, within semi-closed hydroponic strawberry systems, seasonal environmental stimuli—not nutrient recycling practices—are the predominant regulators of antioxidant potential, especially for phenolic content and overall antioxidant capacity.

Among the treatments, although statistically significant differences were not always observed, fruits from the D70% group occasionally displayed increased antioxidant levels (such as DPPH activity and FRAP) compared to the other treatments. This pattern can be attributed to mild osmotic stress resulting from the gradual accumulation of Na^+^, Cl^−^, and SO_4_^2−^ under elevated recycling conditions. Salt stress is recognized to stimulate the generation of reactive oxygen species (ROS), potentially leading to oxidative injury in plant cells and triggering the activation of antioxidant defense mechanisms [30,31]. Specifically, salt stress can interfere with the uptake of K^+^ and Mg^2+^ while promoting Ca^2+^ signaling, ultimately enhancing the production of antioxidant compounds. In summary, the periodic increase in antioxidant levels observed under high recycling conditions likely results from a stress-mediated activation of phenolic biosynthesis and radical scavenging responses, though this advantage is counterbalanced by a simultaneous decrease in fruit size and overall yield [32]. Related findings have been documented in previous research, in which moderate salinity (for example, 10 mM NaCl) elevated total phenolic content (TPC) by 6–10% without negatively affecting yield or total soluble solids (TSS) [15]. In strawberries, Keutgen and Pawelzik [33] demonstrated that salt stress reduced fruit weight but preserved TSS and TSS/TA ratios, and increased both antioxidant capacity and concentrations of Na^+^, Cl^−^, and K^+^.

### 2.4. Principal Component Analysis

**Figure 5:**
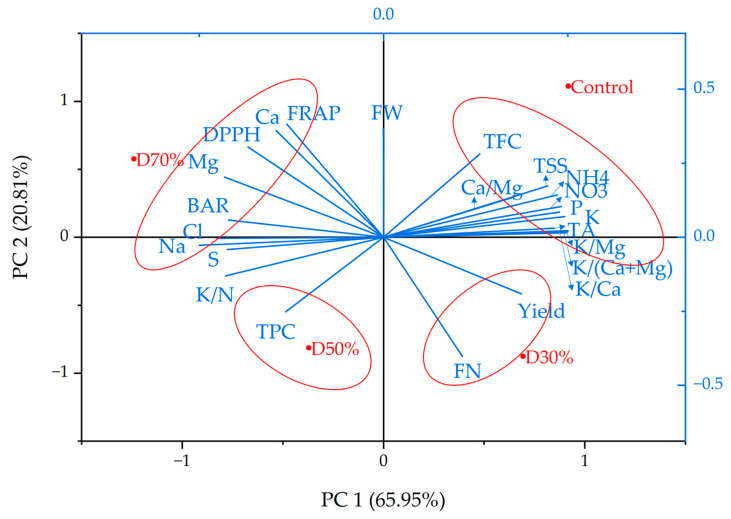
Principal component analysis with strawberry fruit quality parameters, productivity, and inorganic concentration supplied to a closed hydroponic system for strawberry cultivation with different drainage recycling ratios. TPC-total phenol contents; TFC-total flavonid contents; FRAP-ferric reducing antioxidant power; DPPH-2,2-Diphenyl-1-picrylhydrazyl radical scavenging activity; FN-fruit number; FW-fruit weight; TSS-total soluble solids; TA-titratable acidity; BAR-TSS to TA ratio.

Principal component analysis (PCA) was performed to examine the multivariate relationships among fruit quality indicators, antioxidant activity, ion concentrations, and ionic ratios under varying drainage recycling ratios (Figure 5). The first two principal components accounted for the majority of the multivariate variance, with PC1 explaining 65.38% and PC2 explaining 21.10% of the total variance, resulting in a cumulative variance of 86.48%. For PC1, variables representing balanced nutrient status showed positive loadings: NO_3_^−^, NH_4_^+^, and the cation ratios K/Ca, K/Mg, K/(Ca + Mg), and Ca/Mg, along with TSS (and TFC). Conversely, the negative direction of PC1 corresponded to Na^+^, Cl^−^, SO_4_^2−^ (S), the TSS-to-TA ratio (BAR), and the K/N ratio, which are indicators of ionic accumulation and salinity. Accordingly, PC1 arranges the treatments by ascending salinity stress, from Control ≈ D30% → D50% → D70%. PC2 differentiated quality and antioxidant variables from productivity outcomes. Positive loadings on PC2 included FRAP, DPPH, Ca^2+^, Mg^2+^, TFC, and fruit weight (FW), while fruit number (FN) and yield loaded negatively (with TPC also displaying a minor negative trend). This pattern highlights the well-established trade-off between biochemical quality parameters and yield under mineral stress conditions [34].

Treatment positions corresponded to these gradients. Control was located in the upper-right quadrant, adjacent to TSS, NO_3_^−^/NH_4_^+^, and the cation-balance ratios, reflecting low Na^+^ and an intentionally maintained nutrient profile. D30% was positioned in the lower-right quadrant, aligned with the yield and FN vectors, indicating that moderate recycling maintained productivity while mitigating strong salinity effects. D50% was found in the lower-left quadrant near TPC, signifying moderate ion accumulation with a tendency toward elevated phenolic content and partial divergence from the yield vector. D70% was situated in the upper-left, close to Na^+^, Cl^−^, S, BAR, and the antioxidant cluster, demonstrating the most substantial ionic accumulation and a shift toward quality traits (higher TSS/TA and antioxidant capacity) at the cost of productivity.

Overall, the biplot demonstrates a distinct trade-off, where transitioning from Control to D30% maintains or improves productivity with minimal salinity stress, while further increases (D50% → D70%) are progressively associated with ion build-up and quality-driven outcomes. As the PCA was performed using four treatment centroids (n = 4), these findings should be regarded as exploratory ordination; however, the configuration coherently reflects expected semi-closed system salinity behavior and offers targeted, testable hypotheses for subsequent replicated or temporally detailed investigations.

## 3. Materials and Methods

### 3.1. Plant Cultivation and Greenhouse Environment

Korean strawberry plants (cv. ‘Seolhayng’) were grown in an elevated hydroponic system from 20 September 2024 to 31 March 2025, in a smart greenhouse equipped with complex environmental control software (HortiMax, CX500, Ridder, Maasdijk, The Netherlands). Seedlings were transplanted into slab-type coir substrates (L100 × W20 × H12 cm, dust:chip = 10:0 (*v*/*v*), Duck Yang, Sri Lanka) arranged in two rows, each containing seven plants. For each treatment, 10 coir slabs were used, totaling 140 plants. Seedling uniformity was ensured, with each plant having 6.9 leaves and a crown diameter of 12.1 mm. Plant density was 7.2 plants·m^−2^. The greenhouse environment was automatically monitored and regulated by software utilizing temperature and relative humidity (RH) sensors installed inside the facility, and a solar sensor mounted on the greenhouse roof. Environmental data recorded from October to March showed distinct seasonal fluctuations, with the lowest integrated solar radiation (ISR), air temperature, and RH observed from December to February (Table 4). The minimum ISR was detected in December (848 J·cm^−2^). The minimum 24 h-mean temperature occurred in February (13.2 °C), coinciding with the lowest 24 h-mean RH (73.5%) due to heating. In March, environmental conditions shifted toward Spring, showing an increase in ISR (1636 J·cm^−2^), 24 h-mean temperature (16.2 °C), and RH (87.7%).

### 3.2. Experimental Drainage Recycling Ratio Design

**Figure 6:**
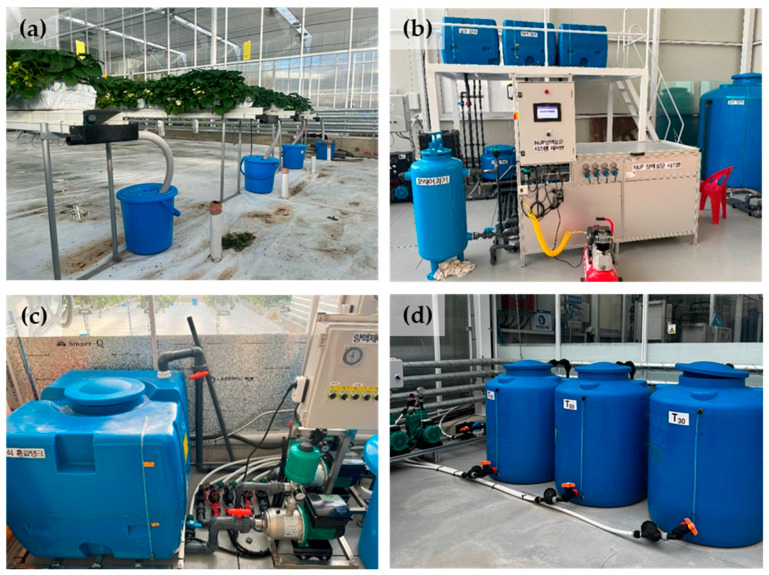
Recycling hydroponic systems used in the experiment: (**a**) drainage containers, (**b**) a nano ultra filtration system with a sand filter, (**c**) a recycling drainage tank, and (**d**) mixing tanks designated for each ratio treatment.

The elevated hydroponic systems operated as semi-closed units with varying drainage recycling ratios (Figure 6). The drainage recycling ratios were set at 30%, 50%, and 70%, based on the electrical conductivity (EC) of the drain. A control group used an open-hydroponic system. The University of Seoul (UOS) nutrient solution (TN-P-K-Ca-Mg-S = 7.3-2.1-2.5-4.5-2.5-2.5 mEq·L^−1^ for the vegetative stage, and 7.0-2.1-4.0-3.0-1.5-1.5 mEq·L^−1^ for the reproductive stage) was supplied at 0.8–1.3 dS·m^−1^ EC and a pH of 5.5–6.0 depending on the growth stage. To minimize the risk of nutritional deficiencies and physiological disorders, 20% mono potassium phosphate (KH_2_PO_4_) was added to the nutrient solution (18 g in 200 L) from 11 January 2025, until the end of cultivation.

Irrigation was automatically managed using a frequency domain reflectometry (FDR) sensor (Coco sensor, Mirae sensor, Seoul, Republic of Korea), maintaining the relative water content of the coir slabs between 66 and 71%, depending on the growth stage, for recycling ratio treatments. Irrigation for the control was regulated by a timer, providing irrigation 4–6 times daily depending on the season. The supply volume was 25 mL/plant for both irrigation strategies, with irrigation beginning at 9 a.m. and concluding three hours before sunset. The delay interval after application for the FDR based irrigation control ranged from a minimum of 30 min to a maximum of 120 min, thereby preventing both overly frequent and inadequate irrigation events.

### 3.3. Ionic Concentration Measurement in Nutrient Solutions

Nutrient solution was sampled each time it was newly prepared in a nutrient solution tank (approximately every 10 days). Each sample was filtered first through filter paper (Whatman No.6, Kent, United Kingdom) and subsequently using a syringe filter (25 mm, 0.45 µm, ANYLAB, Seoul, Republic of Korea) prior to ion chromatography (IC) analysis. The concentrations of macro ions were determined using IC (Eco IC, Metrohm AG, Herisau, Switzerland). Obtained values were expressed as mEq·L^−1^ after conversion from mg·L^−1^.

### 3.4. Yield, Fruit Quality, and Antioxidant Concentration Investigation

#### 3.4.1. Yield and Fruit Quality Investigation

Fruits were harvested biweekly when they reached 90% ripeness, from December 2024 to March 2025. The total number of fruits per slab was recorded, and fruit weight was determined using a digital balance (HS5200S, HANSUNG Instruments Co., Ltd. Seoul, Republic of Korea) after each harvest and then recalculated per square meter based on a plant density of 7.2 plants·m^−2^ (=700 plants/97.5 m^2^).

Sample fruits for quality analysis were selected from 14 sample plants per treatment at the time of harvest. Fruit weights were measured with the digital balance, while fruit length and width were assessed by caliper (Vernier calipers; CD-20CPX, Mitutoyo Corp., Kawasaki, Japan). For TSS and TA analysis, the terminal 1.5 cm of the strawberry fruit was sectioned and juiced. The juice was then analyzed for TSS using a digital sugar meter (ATAGO PAL-1, Atago Co,. Ltd., Tokyo, Japan), and TA was measured in a 50-fold diluted extract using a digital Brix-Acidity meter (PAL-BXIACID4, Atago Co,. Ltd., Tokyo, Japan).

#### 3.4.2. Determination of Fruit Development Period

The fruit development period was evaluated using the third truss from 24 February to 31 March 2025. To monitor enlargement, fruits of uniform size with 10 mm length were tagged with the corresponding date, while for coloration, white fruits that had completed the enlargement phase were also dated. The required number of days was calculated based on the harvesting dates of the marked fruits.

#### 3.4.3. Antioxidant Concentration Measurement

**Figure 7:**
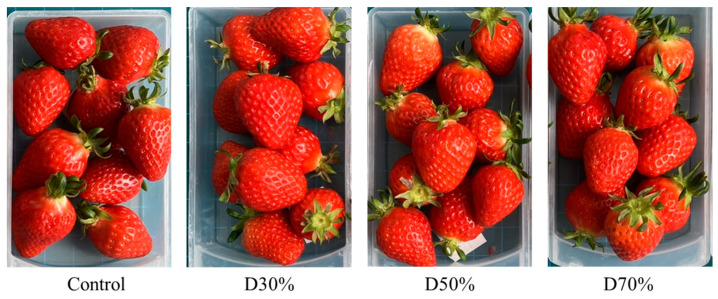
Strawberry fruits sampled for antioxidant analysis. Strawberries were grown in a semi-closed hydroponic system with different drainage reuse ratios for nutrient solution from 17 October 2024 to 14 April 2025.

Fruit samples were collected at approximately 90% ripeness once per month (19 December 2024; 16 January; 17 February; 11 March 2025) (Figure 7). Random selection was performed from 10 coir slabs, with consideration of both size and ripeness to achieve sample uniformity. The average mass of monthly samples was 28.4 g (±1.5), 19.1 g (±0.6), 23.8 g (±2.3), and 26.8 g (±1.0), respectively.

Collected fruit samples were sliced and freeze-dried using a vacuum freeze dryer (HRFD-PMed-BK-EU, Harvest Right, LLC., Salt Lake City, UT, USA). The freeze-dried tissues were ground with a mortar and pestle and stored at −20 °C. For antioxidant extraction, 0.1 g of powdered fruit was combined with 7 mL of 100% methanol, subjected to 30 min of sonication, and centrifuged for 20 min at 4 °C and 4500 rpm (1248R, LaboGene, Gimpo, Republic of Korea) to obtain the first supernatant. After the initial extraction, 3 mL of 80% methanol was added to the pellet, and the mixture was sonicated and centrifuged under identical conditions to collect the second supernatant. Combined supernatants were stored at −20 °C for subsequent analysis.

Total phenolic content (TPC) was determined using a modified Folin–Ciocalteu (F-C) reagent assay [35]. A volume of 200 μL of the total supernatant was mixed with 1250 μL of distilled water and 2.8 mL of F-C reagent. After 3 min, 750 μL of 20% sodium bicarbonate was added. The reaction mixture was incubated for 1 h at room temperature in the dark, and absorbance was read at 765 nm with a UV spectrophotometer (UV-1800, Shimadzu, Kyoto, Japan). TPC was calculated based on a gallic acid calibration curve and expressed in milligrams per gram of dry matter (mg GAE/g DW).

Total flavonoid content (TFC) was measured using a modified version of Chang et al. [36]. For the assay, 200 μL of the total supernatant was combined with 1.5 mL of 100% methanol, 0.1 mL of 10% aluminum chloride, 1 M sodium acetate, and 2.8 mL of distilled water. The resulting solution was incubated in the dark for 30 min at room temperature, then absorbance was recorded at 415 nm using a UV spectrophotometer. Results were quantified using a calibration curve generated with quercetin as the standard, and the total flavonoid content was reported as micrograms per gram of dry matter (μg QE/g DW).

The ferric reducing antioxidant power (FRAP) assay assesses the capacity of antioxidants to reduce ferric ions. The reduced product forms a complex with 2,4,6-tripyridyl-s-triazine (TPTZ), resulting in a change in absorbance at 593 nm. For the assay, 25 μL of supernatant was added to 2.975 mL of FRAP reagent, incubated at 37 °C for 4 min, and absorbance was determined using a UV spectrophotometer. The FRAP reagent consisted of 300 mM acetate buffer, 10 mM TPTZ solution, and 20 mM iron (II) chloride mixed in a 2.5:2.5:5 ratio. Antioxidant power was expressed as mM Trolox equivalent (TE) per 100 g dry matter (mM TE/100 g dry matter).

The DPPH (2,2-diphenyl-1-picrylhydrazyl) radical scavenging activity was evaluated using the Brand-Williams et al. [37] protocol. DPPH reagent was dissolved in 100% methanol, and its absorbance was verified at 1.00 (±0.02) at 517 nm before proceeding. For each assay, 200 μL of sample was combined with 2800 μL of DPPH solution, followed by incubation protected from light at room temperature for 30 min, after which absorbance was recorded at 517 nm with a spectrophotometer. Scavenging activity (%) was calculated according to Equation (1). Abs blank in the equation denotes the DPPH absorbance without sample addition, while Abs sample corresponds to the absorbance post-reaction with the sample.

(1) $$\mathrm{DPPH}\;\mathrm{radical}\;\mathrm{scavenging}\;\mathrm{activity}\;(\%)=(\frac{{Abs}_{blank}-{Abs}_{sample}}{{Abs}_{blank}})\times 100\%$$

### 3.5. Statistical Analysis

Mineral concentrations in the nutrient solutions were averaged by month and treatment, with comparative analysis of mean changes. Yield and fruit count were averaged based on total amount per slab. For fruit quality assessment, weekly measurements were averaged by month and treatment. For antioxidant concentration analysis, ten fruits of uniform size were selected per treatment monthly.

Data were subjected to two-way ANOVA, with month (A) and treatment (B) considered fixed factors. For treatment mean comparisons, one-way ANOVA was performed, and Duncan’s multiple range test was applied as a post hoc test at a significance level of *p* ≤ 0.05. All statistical analyses were conducted using SPSS (SPSS Statistics 29, IBM, Armonk, NY, USA). For further discrimination between treatments and identification of principal factors influencing similarities and differences, principal components analysis (PCA) was carried out with OriginPro 2025 (OriginLab, Northampton, MA, USA).

## 4. Conclusions

This study showed that the drainage recycling ratio strongly influenced nutrient balance and fruit productivity in a semi-closed hydroponic strawberry. Moderate recycling (30–50%) maintained stable K/N and K/Ca ratios, supporting yields comparable to the control, while excessive recycling (70%) caused ionic imbalances, Na^+^ and Cl^−^ accumulation, and reduced fruit size. Antioxidant capacity was more affected by seasonal variation during the cropping period than by recycling level, though higher activity at 70% suggested a mild salt-stress response. Overall, moderate recycling offers an effective strategy to reduce nutrient discharge without compromising yield or fruit quality, whereas higher recycling rates entail a trade-off between environmental benefits and crop performance.

## Figures and Tables

**Table 1:** Monthly mean concentrations of inorganic ion equivalents in nutrient solutions supplied to a closed hydroponic system for strawberry cultivation under varying drainage recycling ratios.

Treatment	NO_3_^−^	NH_4_^+^	PO_4_^3−^	K^+^	Ca^2+^	Mg^2+^	SO_4_^2−^	Total			Na^+^	Cl^−^
								Cation	Anion	C/A		
	--------------------------------------------------------(mEq·L^−1^) --------------------------------------------------------											
December												
Control	7.1 a ^2^	0.6	1.6	3.0	4.6	1.9	2.2	9.4	10.9 ab	0.87	1.5 c	1.5 b
D30%	6.9 a	0.5	1.6	3.0	4.4	1.8	2.6	9.2	11.2 a	0.82	1.8 bc	1.8 a
D50%	5.6 b	0.5	1.3	2.9	4.3	1.8	2.4	9.1	9.4 b	1.08	2.0 ab	1.6 a
D70%	6.0 c	0.5	1.4	3.0	4.3	1.8	2.9	9.1	10.3 ab	0.89	2.2 a	1.9 a
January												
Control	5.5 a	0.7 a	2.8	4.0 a	4.9 a	1.7 a	1.6 c	10.5 a	9.9 a	1.07 bc	1.6 c	1.3 b
D30%	5.1 a	0.3 b	2.3	3.6 ab	4.3 b	1.6 b	2.2 a	9.4 ab	9.6 a	1.03 c	2.3 bc	1.7 a
D50%	2.8 b	0.3 b	1.3	3.0 ab	4.3 b	1.6 b	1.8 bc	9.0 b	5.7 b	1.75 a	2.8 ab	1.3 b
D70%	2.6 b	0.3 b	1.2	2.6 b	4.6 ab	1.7 a	2.0 ab	8.9 b	5.8 b	1.57 ab	3.3 a	1.6 ab
February												
Control	6.4 a	0.5 a	3.8	4.4 a	4.5	1.6	1.8	10.5 a	12.0 a	0.90 b	1.6 d	1.5
D30%	4.7 ab	0.3 b	2.7	4.1 ab	4.6	1.5	1.7	10.2 ab	9.1 ab	1.13 ab	2.0 c	1.5
D50%	3.5 b	0.3 bc	2.1	3.7 ab	4.7	1.7	1.7	10.1 ab	7.3 b	1.53 a	2.7 b	1.5
D70%	3.3 b	0.1 c	2.1	2.9 b	4.7	1.7	2.3	9.3 b	7.7 ab	1.29 ab	3.5 a	2.1
March												
Control	6.7 a	0.6 a	4.1	4.8	4.7	1.7	1.9 c	11.2	12.7	0.89	1.7 b	1.6 c
D30%	6.9 a	0.6 a	4.0	4.4	4.9	1.7	2.1 bc	11.0	13.0	0.84	1.8 b	1.8 c
D50%	5.3 b	0.3 ab	3.5	4.2	5.0	1.7	2.5 ab	10.9	11.2	0.99	3.2 ab	2.3 b
D70%	4.2 b	0.1 b	3.2	4.0	5.3	1.8	3.0 a	11.1	10.5	1.11	4.9 a	2.8 a
Overall mean												
Control	6.5 ± 0.3	0.6 ± 0.0	3.0 ± 0.5	4.0 ± 0.3	4.6 ± 0.1	1.7 ± 0.0	1.9 ± 0.1	10.4 ± 0.3	11.5 ± 0.5	0.9 ± 0.0	1.6 ± 0.0	1.5 ± 0.1
D30%	6.1 ± 0.5	0.4 ± 0.1	2.7 ± 0.4	3.7 ± 0.3	4.6 ± 0.1	1.7 ± 0.1	2.2 ± 0.2	10.0 ± 0.4	11.0 ± 0.8	0.9 ± 0.1	2.0 ± 0.1	1.7 ± 0.1
D50%	4.5 ± 0.6	0.3 ± 0.1	2.1 ± 0.4	3.5 ± 0.3	4.6 ± 0.1	1.7 ± 0.0	2.2 ± 0.2	9.8 ± 0.4	8.8 ± 1.0	1.3 ± 0.2	2.7 ± 0.2	1.7 ± 0.2
D70%	4.3 ± 0.6	0.2 ± 0.1	2.1 ± 0.4	3.2 ± 0.3	4.8 ± 0.2	1.8 ± 0.0	2.6 ± 0.2	9.7 ± 0.4	9.0 ± 0.1	1.2 ± 0.1	3.5 ± 0.5	2.2 ± 0.2
Significance ^1^												
Month (A)	***	***	***	***	n.s.	n.s.	n.s.	***	**	*	n.s.	n.s.
Treatment (B)	**	n.s.	**	**	***	***	**	n.s.	*	*	**	**
(A) × (B)	***	***	***	***	***	***	***	***	*	*	***	***

^1^ n.s.: not significant; *, **, or ***: significant at *p* ≤ 0.05, 0.01, or 0.001 (n = 12). ^2^ Means with different letters are significantly different and without letters are not significantly different according to Duncan’s multiple range test at *p* ≤ 0.05 (n = 3).

**Table 2:** Monthly fruit characteristics of strawberries grown in a closed hydroponic system with different drainage recycling ratios.

Treatment	Fruit Size		TSS	TA	TSS/TA
	Length	Width			
	(mm)	(mm)	(°Brix)	(%)	
December					
Control	49.5 ^2^	39.3	12.8	0.64	21.6
D30%	51.3	42.2	12.7	0.61	21.5
D50%	46.6	38.7	12.6	0.55	22.4
D70%	45.6	39.1	12.3	0.56	22.8
January					
Control	44.9 a	37.7 ab	13.4	0.57	24.8
D30%	43.1 ab	38.2 a	13.1	0.52	26.2
D50%	41.4 bc	36.1 b	13.3	0.55	25.3
D70%	40.4 c	36.1 b	12.8	0.54	24.9
February					
Control	46.7 a	36.9	13.3	0.61 a	22.6
D30%	45.4 ab	36.8	12.7	0.56 b	23.0
D50%	46.0 a	36.6	12.6	0.54 b	23.8
D70%	43.4 b	35.6	12.9	0.56 b	23.5
March					
Control	45.4 ab	33.7	10.5	0.50 b	21.6
D30%	45.7 ab	34.6	11.3	0.65 a	17.9
D50%	46.8 a	34.8	10.5	0.51 b	20.7
D70%	43.5 b	33.3	10.7	0.50 b	21.9
Overall average					
Control	45.9	35.8	12.7	0.57	22.8
D30%	45.1	36.2	12.8	0.54	24.0
D50%	44.6	35.7	12.7	0.57	23.2
D70%	43.2	35.6	12.2	0.58	23.3
Significance ^1^					
Month (A)	**	n.s.	***	***	*
Treatment (B)	n.s.	n.s.	n.s.	n.s.	n.s.
(A) × (B)	**	n.s.	n.s.	*	n.s.

^1^ n.s., not significant; *, **, or ***, significant at *p* ≤ 0.05, 0.01 or 0.001 (n = 4). ^2^ Means with different letters are significantly different and without letters are not significantly different according to Duncan’s multiple range test at *p* ≤ 0.05 (n = 30).

**Table 3:** Monthly antioxidant contents of strawberries grown in a closed hydroponic system with different drainage recycling ratios.

Treatment	Total Phenolic Contents	Total Flavonoid Contents	FRAP	DPPH
	(mg GAE/g DW)	(μg QE/g DW)	(mM TE/100 g DW)	(%)
December				
Control	25.5 ^2^	33.9 a	1.12 b	79.5 b
D30%	24.0	23.0 bc	1.02 b	75.8 b
D50%	23.9	17.8 c	0.98 b	75.8 b
D70%	28.2	24.4 b	1.28 a	88.3 a
January				
Control	26.3 a	23.3	1.17 a	86.4 a
D30%	21.3 b	22.0	1.00 b	77.0 b
D50%	26.0 a	27.2	1.13 a	84.4 a
D70%	25.7 a	28.7	1.18 a	85.1 a
February				
Control	26.7	14.6 b	1.22	88.7
D30%	26.2	25.9 a	1.19	85.9
D50%	25.8	15.2 b	1.16	85.0
D70%	25.1	20.1 ab	1.18	84.2
March				
Control	27.6 b	20.1	1.20 b	48.0 b
D30%	32.5 a	17.6	1.35 ab	57.2 a
D50%	31.5 ab	20.2	1.47 a	55.0 ab
D70%	29.0 ab	15.5	1.32 ab	52.5 ab
Overall average				
Control	26.4	23.2	1.17	77.5
D30%	25.5	22.4	1.13	75.6
D50%	26.5	20.1	1.16	76.4
D70%	26.8	22.6	1.24	79.2
Significance ^1^				
Month (A)	***	**	***	***
Treatment (B)	n.s.	n.s.	n.s.	n.s.
(A) × (B)	***	***	***	***

^1^ n.s., not significant; **, or ***, significant at *p* ≤ 0.01 or 0.001 (n = 4). ^2^ Means with different letters are significantly different and without letters are not significantly different.

**Table 4:** Monthly averages of daily integrated solar radiation (ISR), temperature, and relative humidity measured inside a greenhouse where strawberries were cultivated for fruit quality assessment in 2024/2025.

Month ^1^	ISR	Temperature (°C)					Relative Humidity (%)				
		24 h-Mean	Daytime		Nighttime		24 h-Mean	Daytime		Nighttime	
	(J·cm^−2^/Day)		Ave.	Max.	Ave.	Min.		Ave.	Max.	Ave.	Min.
October	983 ± 153 ^2^	19.6 ± 0.3	23.1 ± 0.8	27.7 ± 1.2	16.8 ± 0.7	14.6 ± 0.8	83.1 ± 0.9	76.2 ± 2.7	64.5 ± 3.1	88.6 ± 0.4	92.0 ± 0.0
November	872 ± 100	17.5 ± 1.1	21.5 ± 1.6	24.3 ± 1.3	14.8 ± 0.7	12.4 ± 0.9	80.4 ± 0.5	72.8 ± 0.9	58.7 ± 4.0	85.6 ± 1.1	86.6 ± 5.8
December	848 ± 31	15.9 ± 0.2	19.3 ± 0.3	22.8 ± 1.4	13.7 ± 0.1	11.6 ± 0.7	78.9 ± 1.8	71.8 ± 3.1	57.7 ± 6.7	83.4 ± 1.0	85.4 ± 6.4
January	885 ± 214	13.7 ± 1.4	17.6 ± 1.3	21.3 ± 1.3	11.0 ± 1.7	8.5 ± 1.9	77.2 ± 4.5	72.9 ± 3.1	57.6 ± 2.9	80.1 ± 5.4	84.4 ± 6.3
February	1241 ± 280	13.2 ± 0.3	17.4 ± 1.0	19.7 ± 2.9	9.7 ± 0.3	6.9 ± 1.1	73.5 ± 3.5	71.9 ± 2.5	50.0 ± 5.6	74.9 ± 4.4	77.9 ± 9.3
March	1636 ± 226	16.2 ± 1.0	20.4 ± 1.1	25.1 ± 3.6	12.1 ± 1.1	9.3 ± 1.7	85.4 ± 8.7	83.0 ± 10.1	66.2 ± 17.0	87.7 ± 7.3	87.6 ± 12.6
Mean	1082 ± 336	16.0 ± 2.4	20.1 ± 2.3	23.1 ± 3.6	13.1 ± 2.6	10.3 ± 2.9	79.4 ± 6.3	74.2 ± 7.2	84.2 ± 10.1	83.1 ± 6.3	57.2 ± 10.0

^1^ The measurement period was from 17 October 2024 to 11 April 2025. ^2^ ± Standard deviation (n = 15 for October; 30 for November; 31 for December, January, and March; 28 for February).

## Data Availability

The original contributions presented in this study are included in the article. Further inquiries can be directed to the corresponding authors.

## Abbreviations

This manuscript includes the following abbreviations:

EC	Electrical conductivity
TSS	Total soluble solids content
TA	Titratable acidity
TPC	Total phenolic content
TFC	Total flavonoid content
FRAP	Ferric reducing antioxidant power
DPPH	2,2-Diphenyl-1-picrylhydrazyl
PCA	Principal component analysis
